# The relationship between homelessness and psychiatric disorders in an inpatient sample

**DOI:** 10.1192/j.eurpsy.2024.1181

**Published:** 2024-08-27

**Authors:** C. V. Vass, G. Gazdag, G. V. Vida

**Affiliations:** Psychiatry and Psychiatric Rehabilitation, Jahn Ferenc South-Pest Hospital, Budapest, Hungary

## Abstract

**Introduction:**

There are more than ten thousand homeless people in Hungary. Earlier studies confirmed the connection between mental health issues and homelessness. Furthermore, homeless care places a significant burden on the healthcare system, with psychiatric departments being no exception.

**Objectives:**

to compare demographic and treatment characteristis of homeless and not homeless inpatients in the psychiatric department of Jahn Ferenc South Pest Hospital (JFSPH), and thus providing a different perspective compared to previous studies, shedding light on the relationships from a different angle.

**Methods:**

In our retrospective study, we analyzed all inpatients’ data treated in the psychiatric department of JFSPH over a 4-month period (03/2023-07/2023). Patients were divided into two groups: those with housing and those who were homeless. We compared the two groups based on the following variables: gender, age, length of inpatient treatment, diagnosis, psychiatric history, employment status; smoking, alcohol and drug use; valid health insurance, invalidity pension and guardianship status; and long-acting injectable antipsychotic treatment.

**Results:**

The percentage of homeless individuals treated in the psychiatric department of JFSPH was 18%. There was a significant difference in the length of inpatient stay between the two groups, homeless patients spent more than 100% longer time under inpatient treatment. Regarding psychiatric history, there was no significant difference between the two patient groups. When examining the employment status of the sample, we found significantly more unemployed patients im the homeless group. Comparing to the control group, there were significantly more alcohol consumers, smokers, and substance users among the homeless. Long-acting antipsychotic injections were administered significantly more frequently to homeless patients. A significantly higher percentage of homeless individuals were declared invalidated, and a significantly higher proportion of them were placed under guardianship compared to the group with housing. Homeless individuals were significantly more likely to have no social insurance compared to the control group.

**Conclusions:**

In summary, we can conclude that significant difference have been found between the homeless and not homeless groups in most of the examined variables. These results implicates that the inpatient care of homeless patients poses significant bourden on the inpatient system. Early prevention and effective rehabilitation of substance use disorders could decrease this burden. Establishing a proper social safety network and enhancing community psychiatric care could potentially also relieve the burden of inpatient care system.

**Disclosure of Interest:**

None Declared

